# Huganbuzure Granule Attenuates Concanavalin-A-Induced Immune Liver Injury in Mice via Regulating the Balance of Th1/Th2/Th17/Treg Cells and Inhibiting Apoptosis

**DOI:** 10.1155/2021/5578021

**Published:** 2021-09-07

**Authors:** Mengheng Wang, Hailong Yin, Yu Xia, Yijun Tu, Xinshuang Zou, Wanci Song, Laichun Luo, Hezhen Wu, Yanfang Yang, Junfeng Zan, Yanwen Liu, Hanxiong Dan, Qiang Yin, Pengtao You

**Affiliations:** ^1^Hubei Key Laboratory of Resources and Chemistry of Chinese Medicine, Hubei University of Chinese Medicine, Wuhan 430065, Hubei, China; ^2^Xinjiang Uygur Pharmaceutical Co., Ltd, Wulumuqi 830001, China

## Abstract

In Uygur medicine, Huganbuzure granule (HBG) is one of the classical prescriptions for liver protection. However, its role in immune liver injury remains unknown. This study evaluates the effect of HBG on concanavalin-A- (ConA-) induced immune liver injury and investigates its protective underlying mechanism. BALB/c mice were randomly divided into five groups (*n* = 24 mice per group): control, ConA, 1.6 g/kg HBG + ConA, 3.2 g/kg HBG + ConA, and 6 mg/kg prednisolone + ConA. HBG was intragastrically administrated once daily for ten consecutive days, prior to ConA (20 mg/kg) injection. The levels of alanine aminotransferase (ALT), aspartate aminotransferase (AST), total bilirubin (TBIL), superoxide dismutase (SOD), and malondialdehyde (MDA) in mouse serum were measured after ConA injection. Moreover, liver-related mRNA levels were evaluated by qPCR. The detection of liver-related proteins was assessed by immunohistochemistry and western blot analysis. Compared with the ConA group, HBG reduced the mRNA expression of IL-17A and IFN-*γ* and the protein expression of T-bet and ROR-*γ*t. In addition, HBG increased the mRNA expression of IL-4 and TGF-*β* and protein expression of GATA3 and Foxp3, indicating that HBG regulated the balance of Th1/Th2 and Th17/Treg. Furthermore, HBG alleviated immune liver injury by reducing oxidative stress, inhibiting apoptosis, and decreasing the expression of p-JNK, p-ERK, p-p38, p-JAK1, p-STAT1, p-STAT3, and IRF1. Our data suggested that HBG attenuated ConA-induced immune liver injury by regulating the immune balance and inhibiting JAK1/STATs/IRF1 signaling, thereby reducing apoptosis induced by JNK activation. The findings indicate that HBG may be a promising drug for immune liver injury.

## 1. Introduction

Immune liver injury is a progressive inflammatory disorder that is closely related to abnormal immune stimulation of liver cells, which may start with acute hepatitis that can eventually result in liver cirrhosis, liver cancer, and even death [1–4]. Although the etiology of immunological hepatic injury is not fully understood, emerging evidence has indicated that, during viral or autoimmune hepatitis, misdirected or excessive immune stimulation may lead to liver injury [5]. CD4^+^ T cells were essential for mediating adaptive immunity in response to a variety of pathogens [6]. Intravenous application of concanavalin A (ConA) in mice resulted in the activation of immature CD4^+^ T cells that subsequently differentiated into *T* helper cells including Th1, Th2, Th17, and regulatory T cells (Treg) [7, 8]. In previous studies, it has been reported that Th1 and Th2 cells play an important role in immunological hepatic injury [9]. Moreover, previous studies have shown that imbalanced Treg and proinflammatory Th17 responses are crucial for the progression of immunological hepatic injury [10, 11]. Furthermore, T cell imbalance has been considered a major cause of immunological liver injury in mice [12].

Apoptosis, as a major constituent of programmed cell death, plays an important role in ConA-induced immune liver injury [13]. Disturbances in the apoptotic process may directly or indirectly be related to the manifestation of a variety of diseases, including tumors and autoimmune diseases. Many factors can induce apoptosis, including radiation and drugs [14]. Several signaling pathways have been associated with the potential mechanism of ConA-induced immune liver injury [15–17].

Uygur medicine, one of the important treatment modalities to prevent and cure diseases in China for thousands of years, plays an important role in ensuring national health and national reproduction. In Uygur medicine, HBG is one of the classical prescriptions for liver protection. It is mainly composed of *Foeniculum vulgare Mill.* (Umbelliferae), *Semen et radix cortex of Cichorium intybus* L. (Compositae), *Cuscuta chinensis Lam.* (Convolvulaceae), and *Semen et radix cortex of Apium graveolens* L. (Umbelliferae). HBG is widely used in the treatment of chronic liver disease [18]; however, the specific underlying mechanisms of HBG in liver protection have yet to be elucidated. The purpose of this study was to establish an immune liver injury model in mice to investigate the underlying mechanism of HBG in maintaining the homeostasis of Th1/Th2 and Treg/Th17 in mice and evaluate the antiapoptosis effects.

## 2. Materials and Methods

### 2.1. Materials

HBG was purchased from Xinjiang Uygur Pharmaceutical Co. Ltd. (Xinjiang, China). Prednisolone was purchased from Shanghai Yuanye Bio-Technology Co., Ltd. (Shanghai, China). ConA was obtained from Sigma-Aldrich (MO, USA). Kits for ALT, AST, TBIL, MDA, and SOD were purchased from the Nanjing Jiancheng Bioengineering Institute (Nanjing, China).

### 2.2. Animal Models and Drug Treatment

A total of 180 male BALB/C mice, six-to-eight weeks old, 20 ± 2 g, were purchased from the Hubei Provincial Center for Disease Control and Prevention (Hubei, China). Mice were housed in an SPF grade environment that was maintained at a temperature of 24 ± 2°C and 55% humidity under a 12 h light/dark cycle with free access to a chow diet and water. All animal experiments were performed based on the National Institutes of Health Guidelines and approved by the Committee on the Ethics of Animal Experiments of the Hubei University of Chinese Medicine. In brief, 120 mice of the experimental group were randomly divided into five groups (*n* = 24 mice per group): control, ConA, 1.6 g/kg HBG + ConA, 3.2 g/kg HBG + ConA, and 6 mg/kg prednisolone (positive control) + ConA. In the HBG and prednisone group, HBG (1.6 or 3.2 g/kg) and prednisone were, respectively, orally administered once daily for 10 consecutive days. The control group and the ConA group received the same volume of normal saline, and the same treatment scheme was followed. The method of animal model construction is based on previously report [19]: on day 11, all mice, except for mice in the control group, were administered 20 mg/kg ConA by tail vein injection. Subsequently, at two, four, and six hours, eight mice from each group were randomly selected and euthanized, and the liver and blood were collected for pathological detection. The same drug treatment schedule was used to determine the survival rate. Survival of 60 mice was closely observed for 12 h after 30 mg/kg ConA injection.

### 2.3. RNA Isolation and Quantitative Real-Time PCR Analysis

Total RNA was extracted from frozen liver tissues using Trizol reagent (Thermo Fisher Scientific, USA), according to the manufacturer's guidelines. RNA was reverse-transcribed using HiScript® III RT SuperMix (Vazyme biotechnology, Nanjing, China). Primers for each gene were designed using Primer Premier 5.0 design software (Premier, Canada) and synthesized by Sangon Biotech (Shanghai, China). The mRNA expression levels of target genes were determined by qPCR using ChamQ Universal SYBR qPCR Master Mix (Vazyme) and the following thermocycler conditions: predegeneration at 95°C for 30 s, 40 cycles of denaturation of 95°C for 10 s, and 60°C for 30 s. This was followed by a melting curve of 95°C for 15 s, 60°C for 60 s, and 95°C for 15 s. To normalize gene expression, GAPDH served as an internal control. The relative quantification was determined by the 2^−ΔΔCT^ method. The primers used for qPCR are listed below (forward primer and reverse primer, respectively): *IFN-γ*, 5′-CTCTTCCTCATGGCTGTTTCT-3′, 5′-TTCTTCCACATCTATGCCACTT-3'; *TNF-α*, 5′-GAGTAGACAAGGTACAACCC-3′, 5′-ACCCTCACACTCAGATCATC-3'; *IL-6*, 5′-CATGTTCTCTGGGAAATCGTGG-3′, 5′-GTACTCCAGGTAGCTATGGTAC-3'; *IL-1β*, 5′-CATCCAGCTTCAAATCTCGCAG-3′, 5′-CACACACCAGCAGGTTATCATC-3'; *IL-4*, 5′-TCTCGAATGTACCAGGAGCCATATC-3′, 5′-AGCACCTTGGAAGCCCTACAGA-3'; *IL-17A*, 5′-GAGCTTCATCTGTGTCTCTGAT-3′, 5′-GCCAAGGGAGTTAAAGACTTTG-3'; *TGF-β*, 5′-TCTGCATTGCACTTATGCTGA-3′, 5′-AAAGGGCGATCTAGTGATGGA-3'; *IFN-β*, 5′-CTGGGTGGAATGAGACTATTGT-3′, 5′-AAGTTCCTGAAGATCTCTGCTC-3'; *GAPDH*, 5′-CATGGCCTTCCGTGTTCCTA-3′, 5′-CCTGCTTCACCACCTTCTTGAT-3'.

### 2.4. Hematoxylin-Eosin and Immunohistochemical Staining

For staining purposes, mouse liver tissues were harvested and fixed in 4% paraformaldehyde for 24 h. Subsequently, the tissue was embedded in paraffin, cut into 5 *μ*m sections, and stained with hematoxylin–eosin (H and E) (Servicebio, #G1005). For immunohistochemical staining, sections were deparaffinized and rehydrated with phosphate-buffered saline (PBS), followed by antigen retrieval. The sections were incubated with peroxidase blocking reagent for 15 min, followed by incubation with 5% BSA for 30 min at room temperature. Next, sections were incubated overnight at 4°C with primary antibodies directed to the following: p-STAT1(1 : 100 dilution, Cell Signaling Technology, #8826), p-STAT3 (1 : 100 dilution, Cell Signaling Technology, #9145), p-JNK (1 : 200 dilution, Cell Signaling Technology, #4668), T-bet (1 : 100 dilution, Absin Bioscience Inc., #abs137236), IRF1 (1 : 100 dilution, Proteintech Group, #11335-1-AP), GATA3 (1 : 500 dilution, Proteintech Group, #10417-1-AP), and Foxp3 (1 : 500 dilution, Proteintech Group, #22228-1-AP). Subsequently, sections were incubated with a secondary horseradish peroxidase- (HRP-) conjugated anti-rabbit IgG antibody (1 : 200 dilution, Cell Signaling Technology, #4412) for 60 min, respectively, and developed using DAB (Servicebio, #G1212) as chromogen. Staining was visualized and images were acquired using a light microscope (Olympus, Japan).

### 2.5. TUNEL Assay

Apoptosis of liver tissues was assessed using the TUNEL assay (Roche, #11684817910). In brief, liver tissues were collected and fixed in 10% buffered neutral formalin at room temperature for at least 24 h and embedded in paraffin. The tissue was cut into 4 *μ*m sections, deparaffinized, and stained with the TUNEL kit. To counterstain, the DAB kit (Servicebio, #G1212) and hematoxylin were used. Sections were observed under a microscope, and images were taken.

### 2.6. Measurement of Reactive Oxygen Species in Liver Cells

The level of reactive oxygen species (ROS) in liver cells was determined using 2,7-dichlorodihydro fluorescent diacetate (DCFH-DA, Beyotime, China), following the manufacturer's guidelines. Sections were observed under a fluorescence microscope (Nikon, Japan), and images were taken.

### 2.7. Western Blot Analysis

For Western blot analysis, liver tissues were collected and homogenized with RIPA lysis buffer, followed by centrifugation at 12,000 rpm to clear the lysates. Next, the protein concentration in the lysates was measured by a bicinchoninic acid (BCA) protein assay. Equal amounts of protein (100 *μ*g) were loaded on an SDS-PAGE gel, separated, and transferred onto nitrocellulose (NC) membranes (Millipore, Burlington, MA, USA). The membranes were blocked with 5% BSA for 2 h at room temperature and then incubated overnight at 4°C with primary antibodies, including ROR-*γ*t (1 : 1000 dilution, Proteintech Group, #13205-1-AP), JAK1 (1 : 1000 dilution, Cell Signaling Technology, #3344), p-JAK1 (1 : 1000 dilution, Cell Signaling Technology, #74129), p-ERK (1 : 1000 dilution, Cell Signaling Technology, #4695), p-p38 (1 : 1000 dilution, Cell Signaling Technology, #9910), and cleaved caspase-3 (1 : 1000 dilution, Cell Signaling Technology, Danvers, #9661). The membranes were washed and then incubated with secondary HRP-conjugated anti-rabbit IgG antibody (1 : 200 dilution, Cell Signaling Technology, #4412) for 1 h at room temperature. The membranes were washed three times with Tris-buffered saline + Tween (TBST) for 10 min. Proteins were visualized by the addition of ECL (Thermo), and membranes were scanned and imaged by the FluorChem FC3 system (ProteinSimple, USA).

### 2.8. Statistical Analysis

Statistical significance was assessed using the Student's *t*-test and one-way ANOVA. The type of *t*-test is two-tailed, equal variance independent-samples *t*-test. All statistical analyses were performed using SPSS 22.0 software (IBM, USA). Data were expressed as the mean ± SD. All experiments were repeated in triplicate, and *p* < 0.05 was considered significant.

## 3. Results

### 3.1. HBG Attenuates ConA-Induced Immunological Liver Injury in Mice

**Figure 1:**
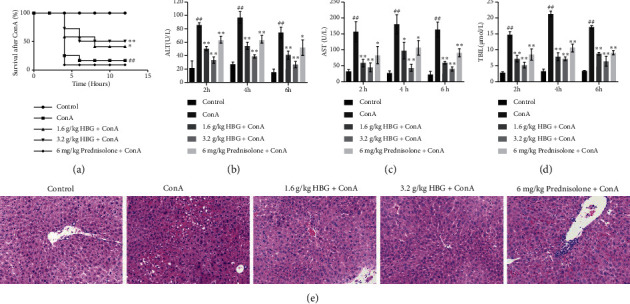
HBG attenuates ConA-induced immunological liver injury in mice. (a) Twelve-hour survival of mice after injection with 30 mg/kg of ConA. (b–d) Serum levels of ALT, AST, and TBIL in serum of mice. (e) H&E staining of mouse liver tissue (×200). Data were expressed as the mean ± SD. ^##^*p* < 0.01 vs. the control group. ^*∗*^*p* < 0.05 and ^*∗∗*^*p* < 0.01 vs. the ConA group.

After the injection of ConA, the survival rate of mice in each group was observed for 12 h. As shown in Figure 1(a), the survival rate of mice in the ConA group was significantly lower compared with that of mice in the control group (*p* < 0.01), indicating that HBG effectively improved the survival rate of mice compared with mice in the ConA group (*p* < 0.05 or *p* < 0.01). Moreover, our data showed that serum levels of ALT, AST, and TBIL significantly increased in the ConA group at all time points and reached a peak at 4 h (*p* < 0.01) (Figures 1(b)–1(d)). However, the increase in ALT, AST, and TBIL expression was reversed by treatment with HBG. Compared with mice in the ConA group, serum levels of ALT, AST, and TBIL of mice that were pretreated with HBG significantly decreased (*p* < 0.01). To further determine the protective effect of HBG on liver injury, H and E staining of liver tissues was performed. Our data demonstrated that in ConA-treated mice, the liver showed massive signs of hepatocyte death, inflammatory cell infiltration, and structural liver damage (Figure 1(e)). However, in the treatment group, only minor slight liver injury was observed. Treatment with HBG, especially, significantly reduced liver necrosis. Taken together, these results showed that HBG effectively reduced ConA-induced immune liver injury in mice.

### 3.2. HBG Inhibits the Production of Inflammatory Cytokines and Regulates Immune Cell Disorder

**Figure 2:**
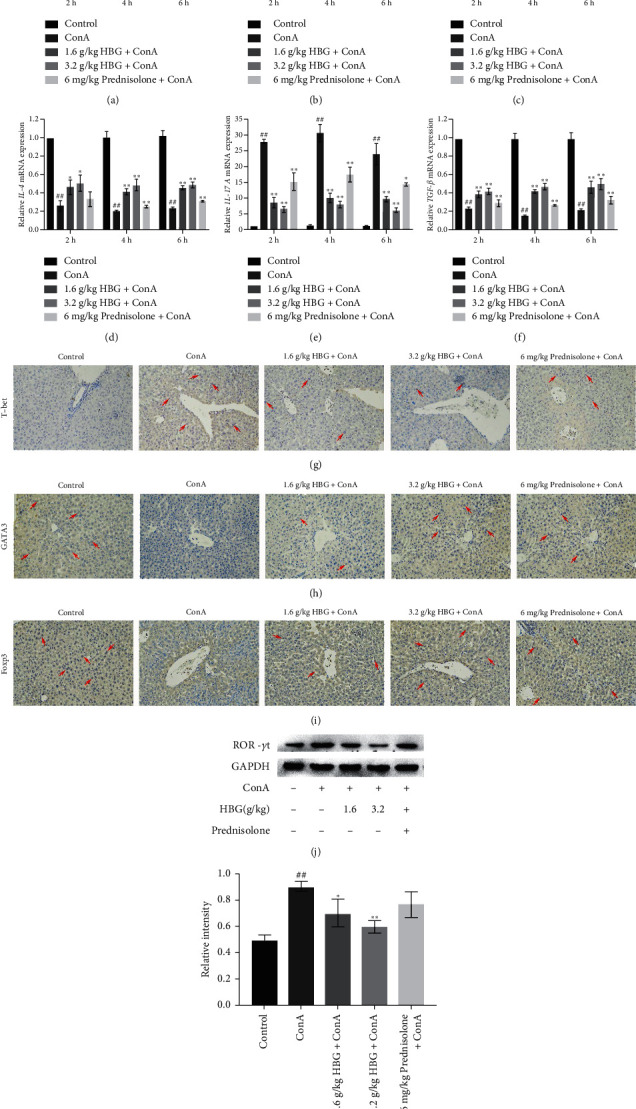
HBG inhibits the production of inflammatory cytokines and regulates the immune cell disorder. (a, b) The mRNA expression levels of IL-1*β* and TNF-*α* were detected by qPCR, normalized to control (2 h) values. (c–f) The mRNA expression levels of IFN-*γ*, IL-4, IL-17A, and TGF-*β* were detected by qPCR, normalized to control (2 h) values. (g–i) Immunohistochemical staining (×200) showing the expression of T-bet, GATA3, and Foxp3 in liver tissue at 4 h after ConA injection. (j, k) The expression of ROR-*γ*t proteins was determined by western blot analysis, and gray values were calculated. ^##^*p* < 0.01 vs. the control group. ^*∗*^*p* < 0.05 and ^*∗∗*^*p* < 0.01 vs. the ConA group.

In general, ConA-induced liver injury is related to the release of inflammatory cytokines [20]. Therefore, qPCR was used to determine the expression of inflammatory factors in mouse liver. As shown in Figure 2, a significant increase in hepatic IL-1*β*, TNF-*α*, IFN-*γ*, and IL-17A was observed at all time points in mice in the ConA group compared with mice in the control group and reached a peak at 4 h after ConA injection (*p* < 0.01). Moreover, the expression of IL-4 and TGF-*β* decreased significantly at all time points and reached the lowest value at 4 h after ConA injection (*p* < 0.01). Treatment with HBG significantly decreased mouse hepatic levels of IL-1*β*, TNF-*α*, IFN-*γ*, and IL-17A mRNA compared with mice in the ConA group (*p* < 0.01) (Figures 2(a)–2(c) and 2(e)) and increased hepatic levels of IL-4 and TGF-*β* (*p* < 0.05 or *p* < 0.01) (Figures 2(d) and 2(f)). Thus, these findings suggested that the hepatoprotective effect of HBG on ConA-induced liver injury in mice may in part be due to the inhibition of inflammatory factors and regulation of immune-related cytokines. Figures 2(g) and 2(j) show that, compared with mice in the control group, the positive expression of T-bet and ROR-*γ*t in mice in the ConA group was significantly increased. Furthermore, expression levels of T-bet and ROR-*γ*t in T cells was reduced in mice in the HBG groups compared with mice in the ConA group. Figures 2(h) and 2(i) show that the positive expression of GATA3 and Foxp3 was significantly reduced after ConA injection. However, treatment with HBG significantly increased the positive expression of GATA3 and Foxp3 compared with that of mice in the ConA group. Taken together, these results suggested that HBG protected against liver injury by inhibiting the production of inflammatory factors and regulating the balance of immune cells.

### 3.3. HBG Suppresses the JAK1/STATs/IRF1 Signaling Pathway

**Figure 3:**
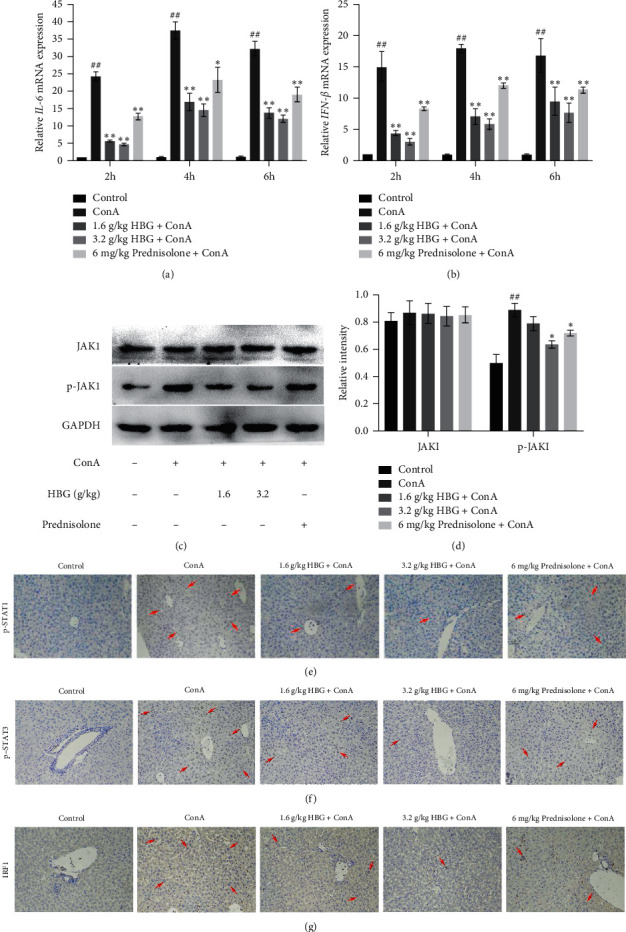
HBG suppresses the JAK1/STATs signaling pathway. (a, b) In each group, mRNA expression levels of IL-6 and IFN-*β* were detected by qPCR, normalized to control (2 h) values. (c, d) The expression of JAK1 and p-JAK1 was determined by western blot analysis, and gray values were calculated. (e–g) Immunohistochemical staining (×200) showing the expression of p-STAT1, p-STAT3, and IRF in liver tissue at 4 h after ConA injection. ^##^*p* < 0.01 vs. the control group. ^*∗*^*p* < 0.05 and ^*∗∗*^*p* < 0.01 vs. the ConA group.

IL-6 can regulate immune responses, acute phase responses, and hematopoietic function and plays an important role in the body's anti-infective immune response [21]. Figures 3(a) and 3(b) show that, at all time points, a significant increase in hepatic IL-6 and IFN-*β* was observed in the ConA group compared with that of mice in the control group and reached a peak at 4 h after ConA injection (*p* < 0.01). HBG significantly decreased hepatic levels of IL-6 and IFN-*β* mRNA compared with that of mice in the ConA group (*p* < 0.01). As shown in Figures 3(c) and 3(d), HBG reduced the p-JAK1 expression in mice with liver injury; however, no significant differences were observed in the expression of total JAK1 between groups. Next, we evaluated the phosphorylation of STAT1 and STAT3 in mouse liver tissues. The data showed that positive expression of p-STAT1 and p-STAT3 was increased in mice in the ConA group compared with mice in the control group. However, HBG effectively downregulated the positive expression of p-STAT1 and p-STAT3, relative to mice in the ConA group (Figures 3(e) and 3(f)). The immunohistochemical data indicated that IRF1 was highly expressed in mice in the ConA group; however, HBG treatment decreased the positive expression of IRF1 to different degrees (Figure 3(g)).

### 3.4. HBG Inhibits Hepatocyte Apoptosis Induced by JNK Activation

**Figure 4:**
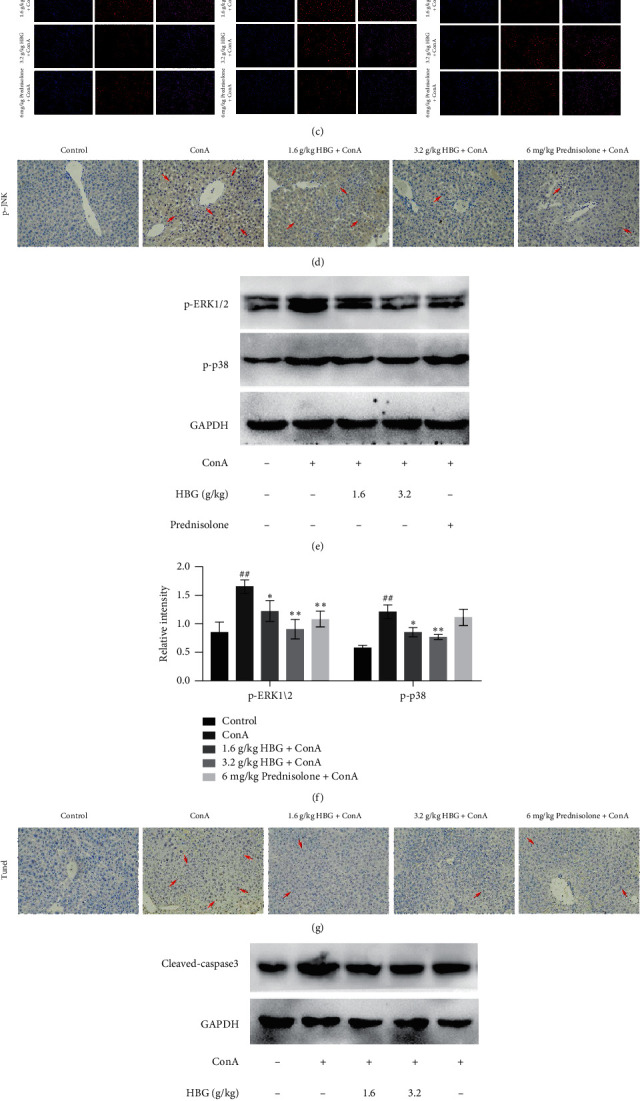
HBG inhibits hepatocyte apoptosis induced by JNK activation. (a, b) Serum levels of MDA and SOD. (c) Reactive oxygen species (ROS) fluorescence staining (×200) showing ROS levels in the liver. (d) Immunohistochemical staining (×200) showing the expression of p-JNK in liver tissue at 4 h after ConA injection. (e, f) The expression of p-p38 and p-ERK1/2 proteins was determined using western blot analysis, and gray values were calculated. (g) TUNEL staining (×200) showing apoptotic cells in five groups at 4 h after ConA injection. (h, i) The expression of cleaved caspase-3 protein was determined using western blot analysis, and gray values were calculated. ^##^*p* < 0.01 vs. the control group. ^*∗*^*p* < 0.05 and ^*∗∗*^*p* < 0.01 vs. the ConA group.

Previous studies have shown that oxidative stress can induce apoptosis [22]. In our study, significant oxidative stress of hepatocytes was observed in mice with liver injury. In brief, we first measured levels of SOD and MDA in mouse liver tissue. The results showed that the MDA content in mice in the ConA group increased at all time points and reached a peak at 4 h after ConA administration (*p* < 0.01). However, administration of 1.6 and 3.2 g/kg HBG effectively inhibited the MDA content at all time points (*p* < 0.05 or *p* < 0.01) (Figure 4(a)). The SOD activity was significantly decreased at all time points in the ConA group and reached the lowest value at 4 h after ConA administration (*p* < 0.01) (Figure 4(b)). Interestingly, the SOD activity was markedly increased by treatment with HBG at all time points (*p* < 0.01). Subsequently, ROS levels in the liver were examined with DCFH-DA staining using frozen liver sections.  Figure 4(c) shows ROS levels at three time points. Compared with the control group, mice in the ConA group showed a significantly higher level of ROS, which reached a peak at 4 h after ConA administration. However, HBG treatment markedly decreased the elevated ConA-induced ROS levels at all time points.

To determine whether p-JNK was involved in ConA-induced liver injury, p-JNK expression was evaluated by immunohistochemical staining.  Figure 4(d) shows that, compared with the control group, significantly increased expression of p-JNK was observed in mice in the ConA group. However, HBG treatment effectively decreased the increased expression of p-JNK. As shown in Figures 4(e) and 4(f), the protein expression of p-ERK and p-p38 in mice in the ConA group was higher compared with that in the control group (*p* < 0.01). HBG significantly downregulated the increase of p-ERK and p-p38 protein expression (*p* < 0.05 or *p* < 0.01).

Simultaneously, we studied the effect of HBG on the apoptosis of mouse liver induced by ConA. TUNEL assay was performed to determine the apoptosis of mouse liver cells.  Figure 4(g) shows a high number of TUNEL-positive hepatocytes existing in mice in the ConA group compared with mice in the control group. Interestingly, TUNEL staining also showed clear improvement of apoptosis in HBG-treated mice. To further explore the effect of HBG on ConA-induced apoptosis of liver cells, we investigated cleaved caspase-3 protein in mouse liver. Our data showed that, compared with mice in the control group, the protein expression of cleaved caspase-3 in mice in the ConA group was significantly increased (*p* < 0.01) (Figures 4(h) and 4(i)). Thus, HBG markedly downregulated the protein expression of cleaved caspase-3.

## 4. Discussion

Immune liver injury is a type of inflammatory disease [20]. The incidence is increasing, and immune liver injury has become an important burden in the world that possesses a serious threat to human life. Our findings demonstrated that HBG effectively protected liver function, regulated immune balance, and inhibited apoptosis caused by oxidative stress, thereby alleviating ConA-induced immune liver injury in mice.

The increase in ALT and AST serum levels caused by hepatocyte division is a sign of pathological damage [23]. HBG significantly decreased ALT and AST levels, as well as causing liver necrosis in mice with liver injury. In the process of immune liver injury, the activation of inflammatory cells starts, which is characterized by direct cytotoxicity or the release of proinflammatory cytokines, thereby inducing liver injury [24, 25]. The protective role of HBG in ConA-induced liver injury has also been related to its strong anti-inflammatory effect.

T lymphocytes play a significant role in immune liver injury and can differentiate into Th1, Th2, Th17, and Treg cells [26]. Intrahepatic tolerogenic immune responses regulate pro- and anti-inflammatory immune balance and maintain intrahepatic balance [27]. However, when this balance is disrupted, excessive immune activation can lead to acute liver failure [5]. Intrahepatic IFN-*γ* is an important cytokine that can induce T-cell-dependent liver injury, is secreted by Th1 cells, and has been reported as a key mediator in the pathogenesis of ConA-induced liver injury [28, 29]. IL-4 is an important cytokine secreted by Th2, and the imbalance of Th1/Th2 has become an important factor in immune liver injury [30]. An imbalance in Treg and Th17 is also a key factor in the development of immune liver injury [31, 32]. Th17 has been recognized as a proinflammatory cell, and IL-17A was secreted by Th17. Our study showed that HBG significantly reduced the expression of IFN-*γ* and T-bet in the liver of ConA-injected mice and, simultaneously, upregulated the expression of IL-4 and GATA3 in the liver. However, HBG significantly reduced the expression of IL-17A and ROR-*γ*t and enhanced the expression of TGF-*β* and Foxp3 in mice with immune liver injury. Together, these findings suggested that HBG reduced immune liver injury by controlling the balance of Th1/Th2 and Th17/Tregs.

Previous studies have shown that IL-6 is an important regulatory factor in the development of autoimmune diseases [33]. IL-6 plays an important role in the differentiation of Th17. The IL-6 content in HBG decreased significantly, which may be the specific initial mechanism of the protective effect of HBG on liver injury. When IL-6 binds to its receptor, JAK kinase, especially, JAK1 and JAK2, promotes phosphorylation of the IL-6 receptor complex, while STAT3 binds to the latter for a short amount of time. Subsequently, STAT3 is phosphorylated by JAK, dissociates into a dimer, and is translated into nucleus. STAT3 plays an important role in cell growth, survival, proliferation, differentiation, and apoptosis [34]. In our study, HBG treatment abolished the increase in p-JAK1 and p-STAT3 in mice with liver injury. Previous studies have shown that the regulation of STAT3 and MAPKs was relative to the modulation of apoptosis [35]. In the development of ConA-induced immune liver injury, the STAT1 pathway in mouse hepatic stellate cells was activated, resulting in the release of IFN-*β*, and the released IFN-*β* activates the STAT1 pathway in hepatocytes, leading to oxidative stress and subsequent apoptosis of hepatocytes [36, 37]. IRF1 is a transcription factor that controls transcription of many antiviral and apoptosis-related genes and is related to the level of oxidation in hepatocytes [36]. It is reported IRF1 can be specifically activated by STAT1 and STAT3 [38]. In our study, treatment with HBG effectively reduced the expression of IFN-*β*, STAT1, and IRF1. Thus, these findings suggested that HBG reduced apoptosis could be related to the inhibition of JAK1/STATs/IRF1 signaling pathway.

Previous studies have shown that JNK was involved in the activation of mitochondria [39, 40]. It has previously been reported that blocking the generation of ROS in the liver almost completely blocked the activation of JNK and decreased the liver damage, suggesting that ROS were the main contributor of JNK activation [41]. Moreover, studies have shown that ConA-induced immunological liver injury decreased significantly with the inhibition of JNK phosphorylation [42–44]. JNK may promote cell death through different mechanisms in different cells. SOD is an important mediator of the primary line of defense against the harmful effects of ROS [45]. In addition, oxidative stress has been considered the main factor of ConA-induced liver injury, which can damage DNA, induce apoptosis, and promote the production of inflammatory cytokines. MDA, a residual product of lipid peroxidation, is often used to indicate the oxidative status of a cell [46]. In this study, we observed that treatment with HBG significantly inhibited the production of ROS and MDA and increased the activity of SOD in mice with liver injury. Furthermore, the protein expression of p-JNK, p-ERK, p-p38, and cleaved caspase-3 was reduced by HBG treatment. Taken together, our findings indicated that the protective effect of HBG on ConA-induced immune liver injury was related to the MAPK family, and that HBG reduced active oxygen and restored antioxidant enzymes of the body to decrease the activation of JNK, thereby inhibiting apoptosis.

## 5. Conclusions

**Figure 5:**
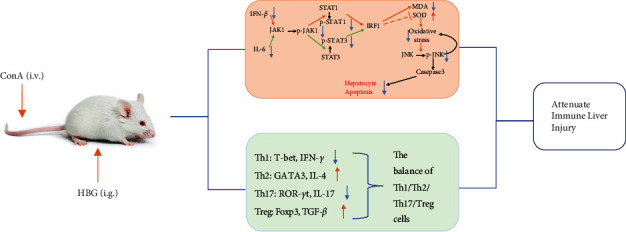
Schematic illustration of the protective mechanism of HBG on immune liver injury in mice.

The protective effect of HBG involved regulating the immune balance and inhibiting JAK1/STATs/IRF1 signaling, thereby reducing apoptosis induced by JNK activation (Figure 5). These findings suggest that HBG may be a promising potential therapeutic agent for immune liver injury. Considering that the chemical substances of HBG are complex, additional studies are essential to elucidate the specific chemical components of HBG that play a therapeutic role in immune liver injury.

## Data Availability

The datasets used and/or analyzed during the current study are available from the corresponding author upon reasonable request.
